# Current progress of ferroptosis in cardiovascular diseases

**DOI:** 10.3389/fcvm.2023.1259219

**Published:** 2023-10-24

**Authors:** Jie Zhang, Caixia Guo

**Affiliations:** Cardiovascular Center, Beijing Tongren Hospital, Capital Medical University, Beijing, China

**Keywords:** cardiovascular disease, ferroptosis, iron metabolism, lipid peroxidation, amino acid metabolism

## Abstract

Ferroptosis, a newly recognized form of nonapoptotic regulated cell death, is characterized by iron-dependent lipid peroxidation. Biological processes, such as iron metabolism, lipid peroxidation, and amino acid metabolism, are involved in the process of ferroptosis. However, the related molecular mechanism of ferroptosis has not yet been completely clarified, and specific and sensitive biomarkers for ferroptosis need to be explored. Recently, studies have revealed that ferroptosis probably causes or exacerbates the progress of cardiovascular diseases, and could be the potential therapeutic target for cardiovascular diseases. In this review, we summarize the molecular mechanisms regulating ferroptosis, inducers or inhibitors of ferroptosis, and the current progresses of ferroptosis in cardiovascular diseases. Furthermore, we discuss the emerging challenges and future perspectives, which may provide novel insights into the treatment of cardiovascular diseases.

## 1. Introduction

Cardiovascular diseases (CVDs), a group of diseases, are currently the leading causes of death globally, imposing a significant economic burden on families and communities (1, 2). Therefore, understanding the pathophysiological progression of CVDs and related molecular mechanisms has become the main focus of medirelated cal studies.

Multiple types of cell death, including apoptosis, necrosis, and pyroptosis, are associated with the pathogenesis of CVDs (3). Accumulating evidence has demonstrated that ferroptosis, a newly recognized type of nonapoptotic regulated cell death (RCD), plays a central role in CVDs, which is triggered by iron-dependent lipid peroxidation (3, 4). Ferroptosis was first reported in 2012, with features of loss of mitochondria crista, cell swelling, and outer membrane rupture, without the change of nucleus (5).

In this review, we focus on effects of ferroptosis and related mechanisms in the occurrence and development of CVDs, and highlight the effective strategy for targeting ferroptosis in CVDs.

## 2. Cellular mechanisms of ferroptosis

**Figure 1:**
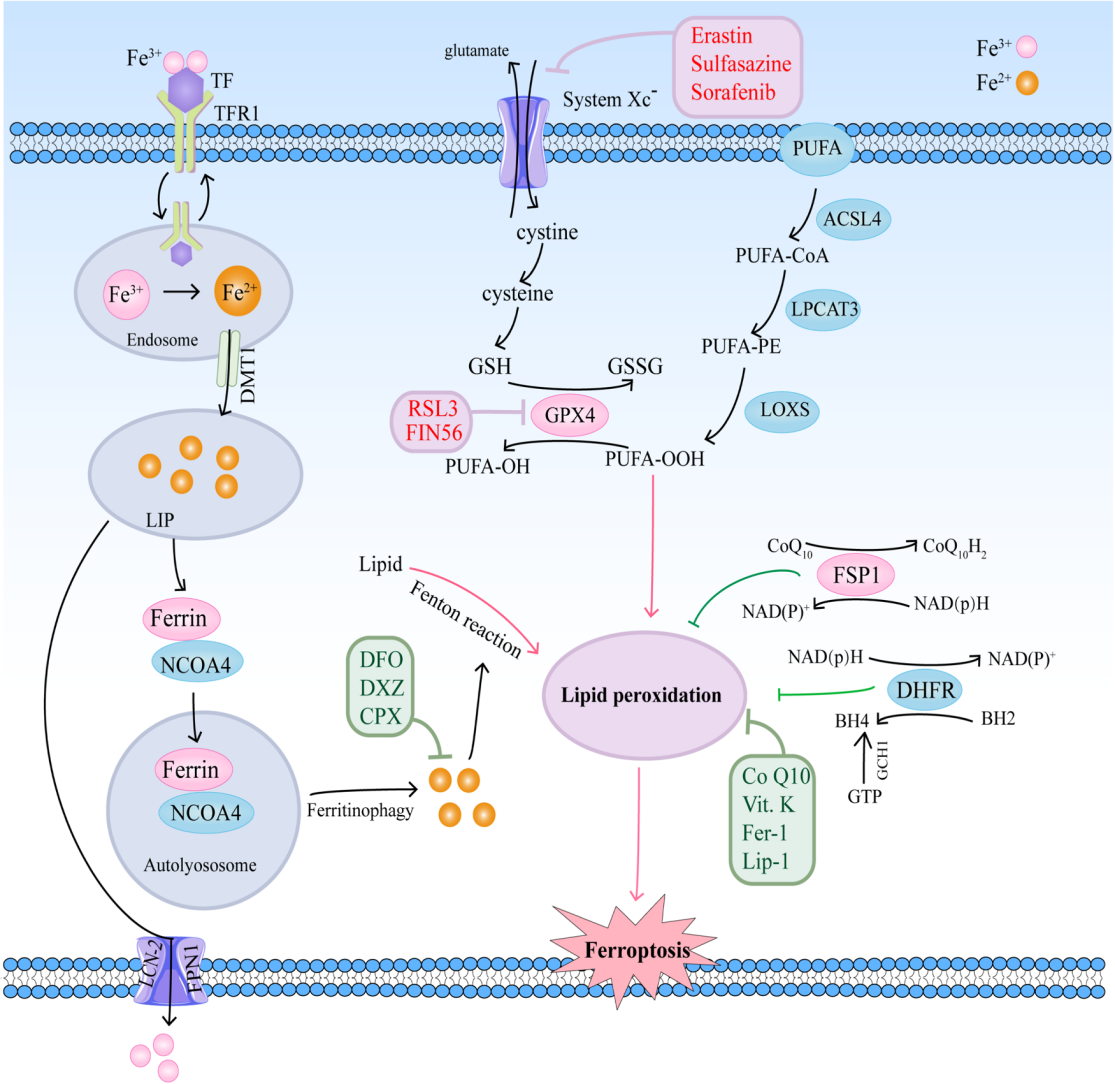
The regulatory mechanisms of ferroptosis in cardiovascular diseases.

Ferroptosis is a complicated process that is moderated by a series of signaling and molecules. The occurrence and execution of ferroptosis contain three main events, iron dysregulation, GSH depletion, and lipid membrane oxidation (6). The regulatory mechanisms of these events and their roles in cardiovascular diseases are summarized in Figure 1.

### 2.1. Iron dysregulation

Iron homeostasis disorders are involved in the pathological processes of cardiovascular diseases. Iron as a necessary cofactor for several enzymes is essential for normal physiological activities, including cell oxygen transport, oxidation–reduction reactions, and energy metabolism (7). Circulating iron is present in two ionic forms, ferrous (Fe2^+^) and ferric (Fe3^+^) iron (8). In general, iron levels in different tissues are tightly regulated, which are accompanied by precise regulation of iron storage protein ferritin and other proteins. Extracellular iron binds to ferritin transferrin (TF) in the form of ferric iron, and is transported into cells by a cell-surface receptor, transferrin receptor 1 (TFR1) (8, 9). Six-transmembrane epithelial antigens of the prostate 3 (STEAP3) catalyzes the reduction of Fe3^+^ to Fe2^+^, and the transporter divalent metal transporter (DMT1) transports Fe2^+^ into the iron pool (LIP) through SLC11A2 (10, 11). The surplus Fe2^+^ is temporarily kept in the ferritin protein, composed of the ferritin light chain (FTL) and ferritin heavy chain 1 (FTH1), or enters into the blood by ferroportin-1 (FPN) and lipocalin-2 (LCN-2) (12, 13).

FPN, the sole exporter in charge of the efflux of surplus iron, is degraded after being bound to hepcidin (14). Accordingly, hepcidin-FPN axis is essential for maintaining iron homeostasis. Altered FPN expression could cause iron deficiency or overload. Knockdown of FPN promotes ferroptosis with an accumulation of intracellular iron and the production of reactive oxygen species (ROS) by the Fenton reaction (13). High level of hepcidin is induced via iron overload and chronic inflammation. Hepcidin binds to FPN and leads to degradation of FPN, thereby causing intracellular iron restriction (15). In contrast, low level of hepcidin in response to iron deficiency or hypoxia stabilizes FPN, leading to more liberation of the iron (15).

Under pathological conditions, intracellular iron homeostasis is disrupted, including increased iron intake or decreased iron stores, which leads to increased Fe^2+^ concentration in extracellular space, and catalyzes the Fenton reaction to participate in the production of ROS, inducing lipid peroxidation and eventually ferroptosis (16). Recent studies have suggested that iron-bound ferritin could bind to nuclear receptor coactivator 4 (NCOA4), promoting the NCOA4-mediated autophagy pathway (namely ferrotinophagy) and resulting in lysosomal degradation of ferritin and iron release (17, 18). Upregulation of NCOA4 increases ferritin degradation and promotes ferroptosis (19). Therefore, both iron overload and ferritin deficiency could trigger ferroptosis.

### 2.2. Lipid peroxidation

Lipid peroxidation is a way that oxidants, including free radicals or non-radical species, attack lipids containing two or more double hydrocarbon chains on the carbon chain. Especially, polyunsaturated fatty acids (PUFAs) are more prone to lipid peroxidation under oxidative stress (20, 21). Cell membranes contain a large number of PUFAs that are primary targets of the attack by ROS (21). Lipid peroxidation can be non-enzymatically and enzymatically triggered. The former process is driven by free radicals, and the latter by enzymes, such as lipoxygenases (LOXs) (20).

PUFAs can be acylated by acyl-CoA synthetase long-chain family member 4 (ACSL4) to form PUFA acyl-coenzyme A (PUFA-CoA), and then reacts with lysophosphatidylcholine acyltransferase 3 (LPCAT3) to form PUFA-PE. PUFA-PE is subsequently oxidized by lipoxygenases (LOXs) to generates PUFAs-OOH (22, 23). Peroxidation of PUFA-PEs mainly depends on ACSL4 and LOXs. Thus, suppressed ACSL4 or LOXs could effectively inhibit ferroptosis. A study with human haploid cells has shown that downregulation of ACSL4 and LPCAT3 inhibits ferroptosis by limiting oxidation-sensitive fatty acids in cell membranes (24). Another study has also demonstrated that ACSL4 is a biomarker associated with ferroptosis sensitivity by regulating lipid metabolism (25).

Several reports have also shown that LOXs, the 15-LOX-1 isoform, especially are a key regulator of lipid peroxidation of ferroptosis (22, 26). Suppression of 15-LOX activity, but not cyclooxygenase (COX) and cytochrome P450 (CYP450), effectively inhibits ferroptosis (22). In addition, a study has indicated that 12-LOX is indirectly upregulated by p53, promoting its ability to oxygenate PUFAs upon ROS stress (27). Therefore, downregulation of certain enzymes (such as ACSL4, LPCAT3, and LOXs) inhibits ferroptosis by reducing lipid peroxidation.

### 2.3. Amino acid metabolism

Ferroptosis has been demonstrated to be controlled by amino acid metabolism. According to study reports, ferroptosis can be induced by erastin, a ferroptosis inducer. Erastin inhibits cystine uptake by the cystine/glutamate antiporter, therefore suppressing antioxidant defenses through GSH reduction (5, 28). The pathway that erastin inhibits cystine uptake is mainly dependent on cystine/glutamate antiporter (system Xc^−^), a membrane amino acid transporter, which consists of the catalytic subunit solute carrier family 7 member 11 (SLC7A11) and the solute carrier family 3 member 2 (SLC3A2) (29). System Xc^−^ transports glutamate and cystine between the intracellular and extracellular at a 1:1 ratio, and reduces the cystine to cysteine, therefore providing the initial materials of GSH in the cell (30). Inhibition of System Xc^−^ could directly decrease cellular cysteine, thus repressing the generation of GSH. Thus, System Xc^−^ plays an important role in the process of ferroptosis-related amino-acid metabolism (31).

In addition, it is widely accepted that GPX4 is another key point in the amino acid metabolic pathway. GPX4 belongs to the selenoprotein family that uses GSH as a reducing agent and turns GSH into oxidized glutathione (GSSG) (32). In the process, PUFAs-OOH is reduced to PUFAs-OH by GPX4, suggesting that GPX4 plays a vital role in inhibiting lipid peroxidation (33). Restricted GPX4 decreases GSSG and increases ROS, thereby inducing ferroptosis (34).

## 3. Regulation of ferroptosis

Lipid peroxidation and iron homeostasis, as well as specific gene interventions, have been shown to regulate ferroptosis. Many molecules in cells, including system Xc^−^ and GPX4, have been implicated in governing ferroptosis (28).

Transcription factors have been shown to impact on the determinants of ferroptosis through regulating the antioxidant system. Kelch-like ECH-associated protein 1 (Keap1)-Nuclear factor E2-related factor 2/antioxidant response element (Keap1-Nrf2-ARE) pathway is the main survival pathway in cells, which protect cells from oxidative and electrophilic insults (35). Nrf2 is considered as a master transcription factor for the antioxidant response. Under normal conditions, Nrf2 protein levels are kept basally low by binding to Keap1, and being then degraded through the ubiquitin-proteasome pathway (36). Under stress conditions, Nrf2 is released from Keap1 and translocated to the nucleus to initiate the transcription of the downstream gene (36). Notably, many proteins and enzymes responsible for the initiation of ferroptosis are the downstream of Nrf2, which are grouped into three broad categories according to their function: iron/metal metabolism, intermediate metabolism, and glutathione synthesis/metabolism (37). Therefore, the Keap1-Nrf2-ARE pathway is central to the ferroptosis.

Another cellular signaling pathway is the ferroptosis suppressor protein 1 (FSP1)–coenzyme Q10 (CoQ10)–nicotinamide adenine dinucleotide phosphate (NADPH) pathway, which is associated with GPX4 and glutathione (GSH) to prevent lipid peroxidation and ferroptosis independently (37). Together with NADPH, FSP1 functions as a CoQ oxidoreductase, and reduces CoQ10 to ubiquinol, a lipophilic radical-trapping antioxidant (RTA), which mediate lipid peroxidation, thereby suppressing ferroptosis (38, 39). These studies suggest that the NAD(P)H/FSP1/CoQ10 pathway is involved in the metabolic processes related to lipid peroxidation.

GTP cyclohydrolase-1 (GCH1)-tetrahydrobiopterin/dihydrobiopterin (BH4/BH2)-phospholipid axis has been shown to be a primary signaling pathway of ferroptosis resistance through upregulation of CoQ10, therefore suppressing oxidative damage and ferroptosis (40). GCH1 overexpression increases BH4/BH2, which could act as membrane-permeant antioxidant and preventing depletion of phospholipids with two PUFAs tails (40). Additionally, GCH1 overexpression does not affect GPX4 and GSH, suggesting that GCH1 inhibit ferroptosis independent of GPX4 (40).

## 4. Ferroptosis inducers and inhibitors

Ferroptosis plays a central role in CVDs, and regulating the levels of some key molecules related to ferroptosis may be helpful in treating CVDs (Table 1). Several compounds have been identified to induce or repress ferroptosis (Figure 1).

**Table 1:** Targeting ferroptosis in CVDs.

Disease types	Animal models	Ferroptosis intervention	Effect and mechanism	References
MIRI	MIRI rat model	DFO	Deferoxamine treatment decreased myocardial infarct size and serum CK activity; decreased ACSL4 and MDA levels; upregulation of GPX4.	(51)
	H/R model of cultured cardiomyocytes	Fer-1 or DXZ	Fer-1 or DXZ suppressed lipid peroxides and prevented cell death induced by H/R.	(52)
AS	High-fat diet-fed ApoE^−/−^ mice	Fer-1 or DFO	Fer-1 or DFO decreased lipid deposition and 4-HNE level in plaques; upregulated the expression of FPN and SLC7A11 in macrophages of plaques.	(62)
	ox-LDL-treated RAW264.7 macrophages	Fer-1	Fer-1 reduced ox-LDL-induced foam cells and macrophage ferroptosis; decreased Fe^2+^, LPO and LDH levels; increased GSH, GPX4, FTH1, SLC7A11 and Nrf2 levels.	(63)
	ox-LDL-treated MAECs	Fer-1	Fer-1 increased cell viability and reduced cell death; decreased iron content and lipid peroxidation; upregulated SLC7A11, GPX4, and eNOS levels; downregulated adhesion molecules.	(67)
DIC	DOX treated mice	Fer-1	Fer-1 preserved cardiac function; alleviated pathological changes cardiomyocyte mitochondrial.	(83)
SIC	LPS treated mice	Fer-1 or DXZ	Fer-1 or DXZ increased survival rate; decreased number of inflammatory cells; decreased the level of PTGS2 and lipid ROS.	(116)
Hypertension	Ang II treated mice	Fer-1	Fer-1 inhibited myocardial hypertrophy and remodeling; increased the expression of xCT, GPX4, and Nrf2.	(111)

ACSL4, Acyl-CoA synthase long-chain family member 4; AS, atherosclerosis; angiotensin II (Ang II); DFO, deferoxamine; DXZ, Dexrazoxane; DIC, DOX-induced cardiotoxicity; Fer-1, ferrostatin-1; FTH1, ferritin heavy polypeptide 1; GPX4, glutathione peroxidase 4; LPO, lipid peroxidation; LDH, lactate dehydrogenase; LPS, lipopolysaccharide; MIRI, myocardial ischemia/reperfusion injury; MDA, malonaldehyde; MAECs, mouse aortic endothelial cells; Nrf2, nuclear factor erythroid 2-related factor 2; PTGS2, prostaglandin endoperoxide synthase 2; ROS, reactive oxygen species; SLC7A11, solute carrier family 7 member 11; SIC, sepsis-induced cardiomyopathy.

### 4.1. Ferroptosis inducers

Ferroptosis inducers are compounds that act by inhibiting system Xc^−^ or altering intracellular iron levels. Many ferroptosis inducers has been found, which can be divided into four classes, class I-IV. Class I ferroptosis inducers inhibit cystine uptake through the system Xc^−^, such as erastin and sorafenib. Erastin binds to SLC7A5, which is one of six light chains that bind SLC3A2 to import cystine, decreasing cellular glutathione, and inducing ferroptosis (5, 26, 33). Sorafenib and its analogs, such as SRS13-45, SRS13-60, SRS13-67, and SRS14-98, also inhibit cystine import via system Xc^−^, deplete glutathione and cause lipid ROS accumulation (41).

Class II inducers directly inhibit GPX4 activity, such as RAS-selective lethal 3 (RSL3). Class II inducers do not deplete GSH, but bind to and inactivate GPX4, resulting in the formation of ROS (33). Class III inducers (such as FIN56), newly identified inducers of ferroptosis via chemoproteomic analysis, induce ferroptosis by two different pathways. FIN56 induces GPX4 degradation through post-translational regulation and depletion of coenzyme Q10 and modulation of mevalonate pathway (42). Class IV inducers, including FINO2 (an endoperoxide-containing 1,2-dioxolane), induce ferroptosis via directly oxidizing labile iron or indirectly inhibiting GPX4 activity (43). The mechanisms by which FINO2 causes GPX4 inactivation remain unclear (44).

### 4.2. Ferroptosis inhibitors

A group of small molecules have been identified as ferroptosis inhibitors, which act by iron chelation, inhibiting lipid peroxidation, or eliminating lipid peroxides. Iron chelators, such as deferoxamine (DFO) and dexrazoxane (DXZ), could deplete the cellular iron pool, and promote the excretion of excess Fe^2+^, thereby preventing ferroptosis (20, 45). Ferrostatin-1 (Fer-1) and liproxstatin-1 (Lip-1), two effective inhibitors of ferroptosis, prevent lipid hydroperoxides accumulation due to the reactivity as RTAs instead of the ability as inhibitors of LOXs (45). In addition, studies have shown that liproxstatin-1 and Vitamin E could regulate ferroptosis by binding to 15-LOX with high affinity, inhibiting the 15-LOX enzymatic activity and then suppressing the generation of oxygenated PE (22). Besides vitamin E, reduced vitamin K confer strong anti-ferroptotic function via FSP1-mediated reduction and act as an RTA (46). Reduced CoQ and its analogs, idebenone, also act as an RTA, suppressing lipid peroxidation and ferroptosis via potent free radical scavenging (47).

## 5. Ferroptosis and CVDs

Multiple cell death types are involved in the complicated mechanisms of CVDs. Recently, ferroptosis has been proven to play a central role in CVDs (3). The association between ferroptosis and various CVDs are summarized in the Table 2 and Table 3.

**Table 2:** Effect and mechanism of Ferroptosis in CVDs

Disease types	Animal models	Factors	Effect and mechanism	References
MIRI	MIRI murine model	HO-1	Silencing HO-1 abolished the iron overload in the ER; suppressed lipid peroxides; prevented ferroptosis induced by H/R	(52)
	MIRI rat model; OGD/R model of H9c2 cells	Resveratrol	Resveratrol inhibited ferroptosis; decreased TfR1 level; increased FTH1 and GPX4 levels.	(54)
	MIRI rat model; H/R model of H9c2 cells	USP7	Inhibiting USP7 decreased infarct size in the area at risk and serum CK activity; reduced the necrosis in H9c2 cells; a decreased CPX4 activity and lipid peroxide (ACSL4 and LPO).	(55)
	H/R model of cardiomyocytes cells	ELAVL1	Knockdown of ELAVL1 increased viability of myocardial cells; inhibited the levels of intracellular ROS, iron, LPO, and LDH activity; inhibited Beclin-1 and LC3 levels; increased p62 and GPx4; inhibited cellular iron level, LDH activity and LPO level.	(56)
AS	High-fat diet-fed ApoE^−/−^ mice	Cigarette tar	Cigarette tar enhanced areas of plaques, necrotic cores and less collagen content; increased 4-HNE level in plaques; downregulated the expression of FPN and SLC7A11 in macrophages of plaques.	(62)
	High-fat diet-fed Hamp^−/−^/Ldlr^−/−^ mice	Hepcidin	Hepcidin deficiency increased serum iron; decreased macrophage iron, aortic macrophage activity, and aortic lipid deposition.	(68)
	ox-LDL-treated RAW264.7	IDH1	Inhibiting IDH1 reduced ox-LDL-induced foam cells and macrophage ferroptosis; reduced Fe^2+^ overload, lipid peroxidation, LDH, and glutathione depletion; elevated GPX4, FTH1, SLC7A11 and Nfr2 levels.	(63)
	ox-LDL-treated HCAECs	PDSS2	Overexpression of PDSS2 promoted the proliferation of HCAECs; suppressed the release of ROS, iron content and ferroptosis of HCAECs; increased Nrf2 level.	(66)
DIC	DOX treated mice	GPX4	GPX4 overexpression ameliorated DOX-induced cardiac impairments; reduced lipid peroxidation, fibrosis, and TUNEL+ cells.	(71)
	DOX treated mice	Sirt1	Sirt1 deficiency aggravated cardiac fibrosis; decreased GPX4, GSH and SOD levels; increased Hmox1, MDA, 4-HNE and Fe^2+^ levels; inhibited Nrf2/Keap1 pathway.	(73)
	DOX treated mice, NRCMs and H9C2 cells	EGCG	EGCG pretreatment alleviates cardiomyocyte ferroptosis; decrease iron accumulation, inhibit oxidative stress and abnormal lipid metabolism; upregulates AMPKα2 and promotes TCA cycle activation.	(78)
SIC	LPS treated mice	TMEM43	TMEM43 Knockdown aggravated cardiac inflammation response; increased serum MDA, cardiac MDA, non-heme iron and ferric iron.	(92)
HF	high-salt diet-induced HFpEF mice model	Canaglifozin	Canaglifozin improved blood pressure, cardiac remodeling, and function; decreased concentrations of Fe^2+^, MDA, and the expression of TFR1, ACSL4, 4HNE and NOX4; increased xCT and FTH1 expression.	(99)

ACSL4, Acyl-CoA synthase long-chain family member 4; AS, atherosclerosis; DOX, Doxorubicin; DIC, DOX-induced cardiotoxicity; ER, endoplasmic reticulum; EGCG, Epigallocatechin-3-gallate; Fer-1, ferrostatin-1; FTH1, ferritin heavy polypeptide 1; GPX4, glutathione peroxidase 4; GSH, glutathione; HO-1, heme oxygenase 1; HCAECs, human coronary artery endothelial cells; HF, heart failure; H/R, hypoxia/reoxygenation; IDH1, isocitrate dehydrogenase 1; LPO, lipid peroxidation; LDH, lactate dehydrogenase; LPS, lipopolysaccharide; MIRI, myocardial ischemia/reperfusion injury; MDA, malonaldehyde; MAECs, mouse aortic endothelial cells; Nrf2, nuclear factor erythroid 2-related factor 2; NRCMs, neonatal rat cardiomyocytes; PDSS2, Prenyldiphosphate synthase subunit 2; ROS, reactive oxygen species; SLC7A11, solute carrier family 7 member 11; SIC, sepsis-induced cardiomyopathy; Sirt1, Sirtuin 1; TMEM43, transmembrane protein 43; USP7, ubiquitinspecific protease 7.

**Table 3:** Animal models of ferroptosis related genes in CVDs.

Disease types	Animal models	Effect and mechanism	Reference
MIRI	ALDH2-deficient mice	ALDH2 deficiency enlarged myocardial infarct size, increased CK-MB, LDH and 4-HNE; iron accumulation; MDA production; glutathione depletion; GPX4 inactivation; increased ACSL4 and TfR1; downregulated FTH1and GPX4.	(117)
	Cardiomyocyte-specific alox15 knockout mice	Alox15 deficiency reduced the size of the myocardial infarcts; inhibited cardiac fibrosis; decreased lipid peroxidation products.	(118)
DCM	Cardiomyocyte specific conditional RDH10-knockout mice	Reduction in RDH10 promoted lipid deposition and FFAs uptake; suppression of GPX4, FSP1 and FPN1 in T2DM mice hearts.	(97)
	AMPKa2-KO diabetic mice	AMPKa2 inhibition reduced the expression of NRF2, SLC7A11, Ferritin and CSH; increased MDA level.	(98)
Hypertension	xCT knockout	xCT knockout reduced cardiac pump function; aggravated cardiac hypertrophy and fibrosis in Ang II-treated mice hearts.	(112)

ALDH2, aldehyde dehydrogenase 2; ACSL4, acyl-CoA synthetase long-chain family member 4; Alox15, 15-lipoxygenase; Ang II, Angiotensin II; CK-MB, creatine kinase MB; DCM, diabetic cardiomyopathy; FUNDC1, FUN14 domain containing 1; FFAs, free fatty acids; FSP1, ferroptosis suppressor protein 1; FPN1, ferroportin 1; FTH1, ferritin heavy polypeptide 1; GSH, glutathione; LDH, lactate dehydrogenase; MDA, malondialdehyde; MIRI, ischemia/reperfusion injury; RDH10, Retinol dehydrogenase 10; TfR1, transferrin receptor 1.

### 5.1. Myocardial ischemia/reperfusion injury

Acute myocardial infarction (AMI) is one of the most common high-risk CVDs (48). Timely restoration of the blood supply remains the effective treatment for ischemic myocardium, called reperfusion (49). However, during this process, the myocardium generates a large number of free radicals, further aggravating myocardium injury, which is known as ischemia/reperfusion injury (MIRI) (49). Ferroptosis, characterized by excess iron accumulation and excessive lipid peroxides, participates in the pathogenesis of MIRI (50). Studies have suggested that ferroptosis happens mainly in myocardial reperfusion instead of ischemia, suggesting that ferroptosis intervention may have numerous beneficial effects on the reperfusion phase but not ischemic phase (51).

As a critical endogenous repressor of ferroptosis, GPX4 determines the severity of MIRI. The level of GPX4 is significantly down-regulated in MIRI-injured myocardium, and overexpression of GPX4 attenuates ferroptosis damage in MIRI (52). GPX4 has been suggested to be a critical regulator of ferroptosis in MIRI. Recent studies have reported that iron metabolism balance also plays a crucial role in MIRI. Inhibition of TfR1 reduces iron contents and ROS levels in H9C2 cells, thereby suppressing H/R-induced ferroptosis (53). Furthermore, downregulation of TfR1 and upregulation FTH1 and GPX4 could also attenuate ferroptosis and prevent MIRI in MIRI rat model (54). In addition, knockdown of ubiquitin-specific protease 7 (USP7) inhibits ferroptosis in p53/TfR1 pathway-dependent in H/R-treated H9c2 cells (55). A growing number of studies suggests that heme metabolism also exerts a significant role in ferroptosis. Heme oxygenase-1 (HO-1) level is increased both in the I/R-injured myocardium and cultured cardiomyocytes. HO-1 degrades heme and therefore causes excess Fe2^+^ in endoplasmic reticulum (ER), triggering ferroptosis after H/R in cardiomyocytes (56). Another study has indicated that overexpressed HO-1 inhibits ischemia-reperfusion-mediated ferroptosis via induction of anti-ferroptotic genes, such as Slc7a11 (57). In addition, it has been shown that ferroptosis partly relied on autophagy (58). Embryonic lethal-abnormal vision like protein 1 (ELAVL1), which is transcriptionally regulated by Forkhead box C1 (FOXC1), is reported to contribute to myocardial I/R-induced ferroptosis though increasing autophagy (59).

### 5.2. Atherosclerosis

Atherosclerosis (AS) characterized with arterial wall lipid deposition is the pathological basis for many vascular disorders. AS has been shown to be associated with inflammation, deposition of lipid peroxides and iron (60). Ferroptosis has been shown to regulate AS and plays a vital role in the development of early and late stage (61). Inhibition of macrophage ferroptosis may significantly improve the progression of AS. In ApoE^−/−^ mice, hepcidin-knockdown or SLC7A11-overexpression inhibits plaque macrophage ferroptosis, therefore delaying the progression of atherosclerosis (62). Inhibiting ox-LDL-induced macrophage ferroptosis through reduced Fe^2+^ overload, lipid peroxidation, or elevated GPX4, FTH1, and SLC7A11 expression may alleviate atherosclerosis (63). Endothelial dysfunction is also critical in the onset and progression of AS (64). Iron overload in endothelial cells induces endothelial dysfunction by alleviating the oxidative stress (65). In human coronary artery endothelial cells (HCAECs), an enhanced PDSS2/Nrf2 axis inhibits ferroptosis by decreasing the release of ROS, providing a promising therapeutic target for AS (66). Moreover, in mouse aortic endothelial cells (MAECs), ferroptosis inhibitors could increase SLC7A11 and GPX4 levels, and reduce lipid peroxidation and iron content, therefore alleviating AS (67). Hepcidin, as a key regulator of iron homeostasis, could bind to and causes the degradation of ferroportin, thereby decreasing serum iron contents. Hepcidin deficiency reduces intracellular macrophage iron and proinflammatory phenotype of macrophages, and decreases atherosclerosis in the murine model (68).

### 5.3. Cardiomyopathy

#### 5.3.1. Doxorubicin-induced cardiomyopathy

Doxorubicin (DOX), a chemotherapeutic drug, can cause cardiotoxicity termed doxorubicin-induced cardiomyopathy (DIC). Ferroptosis, a new type of cell death form, has been implicated in DIC mice. Mitochondria-dependent ferroptosis is involved in the pathogenesis of DIC (69). DOX downregulates glutathione peroxidase 4 (GPX4) and induces lipid peroxidation via DOX-Fe2^+^ complex in mitochondria, causing mitochondria-dependent ferroptosis (69). Both *in vitro* and *in vivo*, DOX downregulates GPX4 and NFS1 protein levels, and upregulates acyl-coenzyme A synthetase long-chain family member 4 (ACSL4) level, causing increased lipid peroxidation and ferroptosis (70, 71). In experimental animals, a novel SPATA2/CYLD pathway contributes to DIC by enhancing ferritinophagy through the deubiquitination of NCOA4 (72).

Many factors have been shown to participated in the process of ferroptosis in DIC. For example, Sirt1 exerts cardioprotective effects in DIC by inhibiting ferroptosis through the Nrf2/Keap1 pathway (73). Hydrogen sulfide (H2S) prevents DIC by inhibiting ferroptosis via targeting the OPA3-NFS1 axis (74). In H9c2 cells, resveratrol activates p62-Nrf2 axis to inhibit doxorubicin-induced ferroptosis in cardiomyocytes (75). In neonatal rat cardiomyocytes (NRCMs), exosomal thioredoxin-1 inhibits ferroptosis in DIC by targets mTORC1 signaling (76). In cultured rat aortic adventitial fibroblasts, Elabela blunts doxorubicin-induced ferroptosis by activating the KLF15/GPX4 signaling (77). Epigallocatechin-3-gallate alleviates doxorubicin-induced ferroptosis and cardiotoxicity by increasing AMPKα2 and activating adaptive autophagy both in *in vitro* and *in vivo* (78). In cultured neonatal rat primary ventricular cardiomyocytes (NRVMs), MITOL/MARCH5 inhibiting doxorubicin-induced ferroptosis by maintaining GSH homeostasis (79). In DIC cellular and animal models, disruption of histamine/H1R axis caused ferroptosis and exacerbates DIC likely by modulating STAT3-SLC7A11 pathway (80).

Ethoxyquin is a lipophilic antioxidant widely applied in food preservation, and has been shown to inhibit GPx4-deficient ferroptosis and prevent DIC both *in vitro* and *in vivo* (81). In a cellular and animal DIC model, melatonin alleviates doxorubicin caused mitochondrial damage and ferroptosis by upregulating YAP expression (82). Acyl-CoA thioesterase 1 in the biosynthesis of polyunsaturated fatty acid prevents cardiomyocytes from doxorubicin-induced ferroptosis by altering the lipid composition (83). In doxorubicin-treated mice, Empagliflozin (EMPA) reduces ferroptosis, fibrosis, apoptosis and inflammation through NLRP3 and MyD88-related pathways, causing significant improvements in cardiac functions (84). Therefore, ferroptosis in DIC could be manipulated with various approaches, which might be a therapeutic target.

#### 5.3.2. Others chemical-induced cardiotoxicity

Ferroptosis has been reported to play a vital role in 5-Fluorouracil and other antitumor agents induced cardiotoxicity in cellular and animal levels. 5-fluorouracil (5-FU) is a potent antitumor drug, and its clinical use is limited for severe cardiotoxic effects. ROS and iron homeostasis dependent ferroptosis play a significant role in 5-Fluorouracil-induced cardiotoxicity in H9c2 cardiomyocytes and mice (85). Resveratrol reduces 5-FU-induced cardiotoxicity by attenuating ferroptosis *in vitro* and *in vivo* (86). Trastuzumab (TZM) is generally used for treat breast cancer. DNA damage and ferroptosis are the pivotal mechanisms in its anti-tumor drug cardiotoxicity. Empagliflozin attenuates trastuzumab-induced cardiotoxicity via suppressing DNA damage and ferroptosis (87). Mitophagy receptor protein FUNDC1 and JNK-mediated ferroptosis is involved in paraquat exposure-evoked cardiac and mitochondrial injury (88).

#### 5.3.3. LPS-induced cardiomyopathy

Sepsis-induced cardiac injury may result in lethal consequences, and cardiomyocyte apoptosis, ferroptosis, and inflammation participate in the progress of sepsis-induced cardiomyopathy (SIC). Ferroptosis plays critical role in lipopolysaccharide (LPS)-induced cardiac injury. ICA69 upregulates of STING level, which further results intracellular lipid peroxidation, eventually aggravating ferroptosis and septic heart injury (89). Dex alleviates sepsis-induced myocardial cellular injury by attenuating sepsis-induced increased HO-1 and iron levels, and inhibits ferroptosis by enhancing GPX4 (90). Ferrostatin-1 (Fer-1), a ferroptosis inhibitor, ameliorates LPS-induced cardiac dysfunction partly by TLR4/NF-κB pathway (91). TMEM43 attenuates LPS-induced cardiac injury through ferroptosis inhibition in mice (92). In LPS-induced endotoxemia mouse and cell models, resveratrol regulates the miR-149/HMGB1 axis and inhibits ferroptosis to protect cardiomyocyte from injury (93). Puerarin inhibits the myocardial injury induced by LPS through AMPK-mediated ferroptosis signaling (94). Therefore, regulating ferroptosis is also a therapeutic target in sepsis-induced cardiomyopathy (SIC).

#### 5.3.4. Diabetic cardiomyopathy

Diabetic cardiomyopathy (DCM), a major complication of type 2 diabetes, is a leading cause of heart failure and death in advanced diabetes. Ferroptosis also engages in the pathogenesis of DCM. A recent study has reported that ferroptosis is involved diabetes-induced endothelial dysfunction by activation of the p53-xCT-GSH axis (95). Astragaloside IV (AS-IV) attenuates cardiomyocyte injury through inhibiting CD36 expression, decreasing cellular lipid deposition, therefore suppressing ferroptosis in the models of DCM rats (96). Retinol dehydrogenase 10 (RDH10) metabolism disorder exists in the hearts of mice and the patients with type 2 diabetes mellitus (T2DM) (96). Reduction in RDH10 inhibits GPX4 and FSP1, promoting ferroptosis (97). In the model of T2D mice with DCM, sulforaphane (a Nrf2 activator) ameliorates cardiac ferroptosis in AMPK/Nrf2-dependent way (98). Canagliflozin (Cana), a hypoglycemic drug of SGLT2i, reduces ferroptosis both in DCM mice and cardiomyocytes by regulating iron metabolism and system Xc^−^/GSH/GPX4 axis (99).

### 5.4. Heart failure

Heart failure (HF) is a complex clinical syndrome with high mortality and morbidity (100, 101). Lipid peroxidation and cardiomyocyte ferroptosis have been reported in a rat model of heart failure (102). Since iron homeostasis is essential for the maintenance of normal function of cardiomyocytes, disrupted iron homeostasis contributes to HF. Knockdown of FTH1, which alters cardiac iron homeostasis and increases lipid peroxidation, leads to severe left ventricular (LV) hypertrophy via ferroptosis (103). Both SLC7A11 overexpression in cardiomyocytes and ferroptosis regulator Fer-1 may inhibit high-iron diet-induced severe cardiac damage (103). In a rat model of high-salt diet-induced HF with preserved ejection fraction (HFpEF), iron overloading and lipid peroxidation have been found in the myocardial tissue, as well as the expression of ACSL4, 4-HNE and NOX4 are increased, while GSH content is decreased (102). These changes eventually lead to ferroptosis, which are reversed by sodium-glucose cotransporter 2 inhibitors (SGLT2i) (102). In a transverse aortic constriction (TAC) mouse model, the circular RNA circSnx12 directly regulates iron metabolism via miR-224-5p/FTH1 regulatory network in myocardial cells, suggesting that circular RNA could be a potential therapeutic target for HF (104). Furthermore, expression of Toll-like receptor 4 (TLR4) and NADPH oxidase 4 (NOX4) is significantly increased in HF, and either TLR4 or NOX4 knockdown inhibits ferroptosis by regulating ferroptosis-related proteins, suggesting that TLR4-NOX4 pathway might be a potential therapeutic target for HF (105).

### 5.5. Atrial fibrillation

Atrial fibrillation (AF) is the most prevalent type of cardiac arrhythmia with higher morbidity and mortality, due to stroke, heart failure and other complications (106). Ferroptosis is related to frequent excessive alcohol consumption induced AF (107, 108). Mechanistically, inhibition of atrial ferroptosis via SIRT1-Nrf2-HO-1 signaling to reducing atrial mitochondrial damage, ROS accumulation and iron overload, eventually decrease the AF susceptibility (107). FPN, an iron exporter, is involved in LPS-induced endotoxemia rat model. Knockdown FPN increases intracellular iron concentration, worsens the calcium handling proteins dysregulation and exaggerates the AF vulnerability (109).

### 5.6. Hypertension

Hypertension is a major risk factor for numerous CVDs, and leads to pathological cardiac hypertrophy and eventually heart failure (110). Elabela (ELA), endogenous ligand for the apelin receptor, prevents myocardial remodeling through inhibiting ferroptosis via regulating the IL-6/STAT3/GPX4 signaling in angiotensin II (Ang II)-induced hypertensive mice (111). Moreover, Slc7a11 gene-encoded plasma membrane cystine/glutamate antiporter xCT exerts protective effects in Ang II-medicated cardiac hypertrophy by blocking ferroptosis (112). Ferroptosis is also involved in hypertensive renal injury. Sirtuin 7 alleviates renal ferroptosis and lipid peroxidation via Nrf2/GPX4/xCT and HO-1/NQO1signaling pathways in hypertensive mice (113).

## 6. Limitations and perspectives

Ferroptosis is a newly-identified iron-dependent form of cell death. Recent studies have suggested that ferroptosis could be a feasible therapeutic target for several cardiovascular diseases. However, the molecular mechanism of ferroptosis in cardiovascular diseases has not been fully understood. Reliable and sensitive biomarkers for ferroptosis need to be explored. Most investigations are confined to *in vitro* cell cultures, ex vivo models, and animal studies. The clinical studies relevant to ferroptosis in cardiovascular diseases are urgently needed. Currently, many chemical compounds have been found to affect the process of ferroptosis, including dysregulation of iron metabolism, lipid peroxidation, and abnormal amino acid metabolism. However, selectivity and side-effects of these compounds remain to be examined. It still faces great challenges in translating these compounds as future pharmacological drugs. In addition to ferroptosis, other forms of cell death, such as apoptosis, regulated necrosis and pyroptosis, have also been involved in the pathogenesis of CVDs. For examples, apoptosis and necroptosis are detected in diabetic mouse hearts at different ages (114). Palmitic acid induces the cell death of H9c2 cardiomyoblasts in a dose- and time-dependent manner (115). However, the occurrence timing of those cellular death forms in different CVDs is difficult to determine. The cross-talks may exist between those types of cell death. Therefore, it is unable to provide reliable and accurate information to guide the time window for intervention.

In conclusion, further studies are needed to identify specific biomarkers and central regulators of ferroptosis, providing solid evidence and basis for further exploration in clinical trials. Furthermore, it is important to clarify the different temporal stages of ferroptosis and other cell death forms in CVDs.
